# Predicting neurocognitive function with hippocampal volumes and DTI metrics in patients with Alzheimer's dementia and mild cognitive impairment

**DOI:** 10.1002/brb3.766

**Published:** 2017-07-30

**Authors:** Geumsook Shim, Kwang‐Yeon Choi, Dohyun Kim, Sang‐il Suh, Suji Lee, Hyun‐Ghang Jeong, Bumseok Jeong

**Affiliations:** ^1^ KAIST Clinic Pappalardo Center KAIST Daejeon Korea; ^2^ Department of Psychiatry Korea University College of Medicine Seoul Korea; ^3^ Computational Affective Neuroscience and Development Laboratory KAIST Graduate School of Medical Science and Engineering Daejeon Korea; ^4^ KAIST Institute for Health Science and Technology KAIST Daejeon Korea; ^5^ Department of Radiology Korea University Guro Hospital Korea University College of Medicine Seoul Korea; ^6^ Department of Biomedical Sciences Korea University Graduate School Seoul Korea

**Keywords:** Alzheimer's dementia, diffusion tensor imaging, hippocampal volume, mild cognitive impairment, partial least squares regression

## Abstract

**Introduction:**

Cognitive performance in patients with Alzheimer's dementia (AD) and mild cognitive impairment (MCI) has been reported to be related to hippocampal atrophy and microstructural changes in white matter (WM). We aimed to predict the neurocognitive functions of patients with MCI or AD using hippocampal volumes and diffusion tensor imaging (DTI) metrics via partial least squares regression (PLSR).

**Methods:**

A total of 148 elderly female subjects were included: AD (*n* = 49), MCI (*n* = 66), and healthy controls (*n* = 33). Twenty‐four hippocampal subfield volumes and the average values for fractional anisotropy (FA) and mean diffusivity (MD) of 48 WM tracts were used as predictors, CERAD‐K total scores, scores of CERAD‐K 7 cognitive subdomains and K‐GDS were used as dependent variables in PLSR.

**Results:**

Regarding MCI patients, DTI metrics such as the MD values of the left retrolenticular part of the internal capsule and left fornix (cres)/stria terminalis were significant predictors, while hippocampal subfield volumes, like the left CA1 and hippocampal tail, were main contributors to cognitive function in AD patients, although global FA/MD values were also strong predictors. The 10‐fold cross‐validation and stricter 300‐iteration tests proved that global cognition measured by the CERAD‐K total scores and the scores of several CERAD‐K subdomains can be reliably predicted using the PLSR models.

**Conclusions:**

Our findings indicate different structural contributions to cognitive function in MCI and AD patients, implying that diffuse WM microstructural changes may precede hippocampal atrophy during the AD neurodegenerative process.

## 1 INTRODUCTION

Alzheimer's dementia (AD) usually progresses slowly for a decade or more before a diagnosis of dementia, and a mild cognitive impairment (MCI) is proposed to capture the prodromal stages of various etiologies of dementia, including AD. To date, molecular, functional, and structural biomarkers have been developed to accurately diagnose AD (Ishii, 2014) and to predict conversion from MCI to AD at an early time (Forlenza, Diniz, Teixeira, Stella, & Gattaz, 2013). Among these biomarkers, structural MRI is indispensable, and atrophy of the medial temporal lobe including the hippocampus is considered as a valid diagnostic marker (Frisoni, Fox, Jack, Scheltens, & Thompson, 2010) and as a risk factor of conversion to AD (Grundman et al., 2002). A link between the hippocampal volume and cognitive function, such as verbal memory and language, has been reported in AD, amnestic MCI, and the normal elderly population (Chetelat et al., 2003; Petersen et al., 2000), and hippocampal atrophy is generally accepted as being correlated with cognitive performance in AD.

Compared to hippocampal atrophy, white matter (WM) alterations have not received much attention until recently, since cognitive performance has been considered to be associated with gray matter atrophy rather than with WM alterations (Grundman et al., 2002; Loewenstein et al., 2009). However, a recent meta‐analysis of diffusion tensor imaging (DTI) revealed that microstructural alterations in WM in MCI and AD are widespread throughout the brain (Sexton, Kalu, Filippini, Mackay, & Ebmeier, 2011), particularly in limbic fibers connected directly to the medial temporal lobe (Salat et al., 2010). Subtle impairments in nonmemory performance in MCI patients are postulated to be mainly related to pathology outside the hippocampus (Grundman et al., 2003), and disruption of parahippocampal WM fibers contributes to memory decline by partially disconnecting the hippocampus from incoming sensory information (Stoub et al., 2006).

Two main diffusion metrics are generally calculated from DTI data: fractional anisotropy (FA) and mean diffusivity (MD). FA and MD measure the directional dominance and overall degree of water diffusion in tissue, respectively. FA is generally interpreted as reflecting the density of nerve fibers and their myelin sheaths (Beaulieu, 2002), while MD reflects the breakdown of tissue cytoarchitecture and demyelinating process (Le Bihan & Johansen‐Berg, 2012). Aging and WM neuropathology often result in a decrease in anisotropy, and age‐related FA decline is correlated with slower responses in the visual task (Lebel et al., 2012; Madden et al., 2004).

In the hypothetical model of dynamic biomarkers related to the AD pathological cascade, the production of soluble amyloid beta (Aβ) oligomers, which can directly injure WM integrity (Lee et al., 2004; Roth, Ramirez, Alarcon, & Von Bernhardi, 2005), happens first, and neurodegenerative biomarkers such as cerebral atrophy, synaptic dysfunction, and cognitive symptoms are manifested later (Jack et al., 2010). According to the neurovascular hypothesis of AD, vascular‐derived insults are considered to initiate neuronal degeneration. That is, cerebral hypoxia and blood–brain barrier leakage, caused by the vascular risk factors lead to the accumulation of neurotoxic molecules and WM micro‐injury (Zlokovic, 2011). Vascular injury also hampers the normal clearance of Aβ, leading to the accelerated accumulation of Aβ. Therefore, microstructural changes in WM can be a good biomarker for both early diagnosis of AD and to monitor disease progression (Oishi & Lyketsos, 2016).

Several researches have reported that DTI metrics are associated with several domains of cognitive function in patients with MCI or AD. Mielke and colleagues (Mielke et al., 2012) found that fornix FA is correlated with cross‐sectional memory and predicts memory decline in MCI patients. In studies using linear regression analysis, parietal or temporal lobe FA values and the mean MD values from cinguli were significant predictors for global cognition or episodic memory of amnestic MCI and AD (Bozzali et al., 2012; Wang et al., 2013). However, above‐mentioned studies extracted DTI measures from a priori selected brain areas such as fornix and cinguli, and examined the predictability using linear regression analysis or multivariate model. In a multiple regression model, collinearity, a phenomenon in which two or more predictors are highly correlated, can affect the calculations for individual predictors and distort the interpretation of a model (Tu, Kellett, Clerehugh, & Gilthorpe, 2005; Wold, Ruhe, Wold, & Dunn, 1984). Unfortunately, variables from neuroimaging data are usually quite numerous and are likely to be collinear.

Partial least squares regression (PLSR) combines features from and generalizes principal component analysis and multiple linear regression (Abdi, 2010), and it is particularly useful when we need to predict a set of dependent variables from numerous, highly collinear independent variables or predictors (Tobias, 1995). This prediction is achieved by extracting a set of orthogonal factors from the predictors (i.e., latent variables, LVs) explaining the covariance between predictors and dependent variables as much as possible with the best predictive power (Abdi, 2010).

In this study, we aimed to predict neurocognitive functions of patients with MCI or AD using hippocampal volumes and DTI metrics with PLSR. We hypothesized that several variables among hippocampal subfield volumes and DTI metrics, such as FA and MD, might significantly contribute to the cognitive functions of both patients, but DTI measures might be more predictive for cognitive function in MCI patients compared to AD patients since microstructural changes in WM were considered to precede hippocampal atrophy.

## 2 METHODS

### 2.1 Subjects and imaging data

The potential patients were recruited from the Psychiatric Department, Korea University Guro Hospital, and control subjects were sought from the community population via an advertisement. All subjects received a Korean version of the Consortium to Establish a Registry for AD Assessment Packet (CERAD‐K) (Lee et al., 2002), Korean Geriatric Depression Scales (K‐GDS) (Bae & Cho, 2004), the Korean version of the Clinical Dementia Rating (CDR) scale (Choi et al., 2001). The CERAD Neuropsychological Battery (CERAD‐NB) was developed as a reliable, standardized battery to measure primary cognitive manifestations of AD (Morris et al., 1989). The original CERAD‐NB included five cognitive tests: Verbal Fluency, 15‐item Boston Naming Test, Mini‐Mental State Examination (MMSE), 10‐item Word List Learning, Recall and Recognition Test, and Constructional Praxis and Constructional Recall. The CERAD total score was calculated as the sum of the scores of CERAD subdomains except for MMSE and Constructional Recall, as previously described (Chandler et al., 2005).

The AD diagnosis was made based on the criteria for probable or possible AD, developed by the National Institute of Neurological and Communicative Disorders and Stroke and the Alzheimer's Disease and Related Disorders Association (NINCDS‐ADRDA)(McKhann et al., 1984). Individuals were categorized as MCI based on the criteria proposed by Peterson and colleagues (Petersen et al., 1999). Healthy controls (HCs) met the following criteria: a MMSE score >−1.5 SD (adjusted for age, sex, and educational years) and no objective cognitive impairment (all scores of CERAD‐K cognitive domains >−1.5 SD).

### 2.2 MRI image acquisition

All subjects underwent an MRI examination at the Brain Imaging Center, Korea University. Multiple diffusion‐weighted images, with 20 encoding directions and an additional T2‐weighted scan, were acquired twice at a single‐scan session using a 3.0‐T Siemens Trio Tim scanner with a standard single‐shot, spin echo, echo planar acquisition sequence with eddy current balanced diffusion weighting gradient pulses to reduce distortion (Reese, Heid, Weisskoff, & Wedeen, 2003). The scan parameters were: *b* = 1000 s/mm^2^, TE/TR = 84 ms/6.3 s; matrix = 128 × 128 on 230 × 230 mm FOV; 3‐mm slices without a gap resulting in voxels of 1.8 × 1.8 × 3.0 mm. Four magnitude averages provided sufficient signal‐to‐noise ratios. Volumetric T1‐weighted anatomic reference images were acquired using a magnetization‐prepared rapid gradient echo sequence (TE/TR/TI = 2.60 ms/1.9 s/900 ms; 256 × 256 × 176 matrix for 0.86 × 0.86 × 1 mm voxels).

A total of 60 AD patients, 93 MCI patients, and 48 HCs participated in the MRI examination, and the gender ratios were as follows: (F:M) AD 49:11, MCI 66:27, HCs 33:15. After considering significant differences between the genders in the total intracranial volume (ICV) (*F*, 1319 ± 103; M, 1512 ± 110, *t*‐value = −11.51, *p* < .001) and a relatively small proportion of male subjects (26%), only female subjects were included into this study.

### 2.3 Image preprocessing: hippocampal subfield volumes

Individually, 24 hippocampal subfields were automatically segmented on the T1‐weighted images using Freesurfer v6.0 (Iglesias et al., 2015), which provides volumes for Cornu Ammonis (CA) regions 1, 2 and 3 combined, and 4 (CA1, CA2/3, and CA4), fimbria, hippocampal fissure, presubiculum, subiculum, hippocampal tail, parasubiculum, granule cell layer‐molecular layer‐dentate gyrus (GC‐ML‐DG), molecular layer, and the hippocampus‐amygdala‐transition‐area (HATA).

### 2.4 Image preprocessing: DTI metrics

Preprocessing for DTI analysis, including skull stripping and Eddy current correction, was performed using the FMRIB Software Library (FSL; Oxford, UK; http://www.fmrib.ox.ac.uk/fsl). A diffusion tensor model was arranged for each voxel with the generation of FA and MD images using FMRIB's diffusion toolbox in FSL. The Tract‐Based Spatial Statistics (TBSS) pipeline was used to identify a common registration target, and all subjects’ FA images were aligned to this target using nonlinear registration. The aligned FA images were affine transformed into 1.0 × 1.0 × 1.0 mm^3^ Montreal Neurological Institute 152 space, and a mean FA image was generated from the transformed FA images. A mean skeleton image was created from the mean FA image, and each of the subject's aligned FA image was projected onto the mean FA skeleton by filling the structure with FA values from the nearest relevant tract center. Finally, this skeletonized FA image was thresholded using an FA value of 0.2 to reduce intersubject variability and to represent each tract as a single line running down the center of the tract. The MD images were projected onto the skeleton using the transformation matrix produced by processing the FA images. The average values for FA and MD were calculated within the TBSS skeleton of 48 WM tracts based on the International Consortium of Brain Mapping DTI‐81 WM labels atlas (Smith et al., 2006).

### 2.5 Cognitive function prediction

PLSR modeling was performed using the package “pls” (Mevik, Wehrens, & Liland, 2016) in the R program (R Core Team, 2017). Two kinds of PLSR model were made for each group, and all models used hippocampal subfield volumes and FA/MD values as predictors. As for dependent variables, one used CERAD‐K total scores, while the other used scores of seven CERAD‐K subdomains and K‐GDS, simultaneously. All predictors and dependent variables were transformed into z‐scores, but they were not adjusted to ICV or age since this study's aim was to test the predictability of the hippocampal volumes and DTI metrics *per se*, probably containing the information of ICV or age, on cognitive function for each group, which is also related with ICV or age (Royle et al., 2013; Wolf, Julin, Gertz, Winblad, & Wahlund, 2004). The optimal number of LVs was determined based on the mean squared error of prediction (MSEP) and the predicted residual estimated sum of squares (PRESS). The number of LVs showing the lowest MSEP and PRESS were selected in the PLSR using CERAD‐K total scores as one dependent variable, and in the case of PLSR using multiple dependent variables, the majority rule was applied, which means that the most common number was selected among the numbers of LVs with the lowest MSEP and PRESS for each of the dependent variables. For all predictors per group, jackknife approximate *t*‐tests of regression coefficients (i.e., jack.test in the package “pls”) were performed to identify important predictors that are significantly correlated with each of the LVs with regard to each of the dependent variables separately. To validate the predictability of each group's PLSR models, a 10‐fold cross‐validation and linear regression analysis between observed and predicted scores was conducted. Finally, we randomly split data into 2/3 and 1/3 for the training and test set and predicted the scores of dependent variables with a PLSR model using training set and then performed a linear regression analysis between the observed and predicted scores for the testing set, which was repeated 300 times.

## 3 RESULTS

The demographic data and scores on the CERAD‐K cognitive test, CDR, and K‐GDS are presented in Table 1. The AD and MCI groups are significantly older than the HCs, and AD patients were less educated compared to the HCs. Naturally, the three groups differed from each other in the scores for the CDR and CERAD‐K cognitive test except for the constructional praxis scores, for which the AD group only differed significantly from the MCI and HC groups.

**Table 1:** Demographic and clinical characteristics of participants

	AD (*n* = 49)	MCI (*n* = 66)	HC (*n* = 33)	*F*‐statistic	*p*‐value
Age	77.9 ± 6.3^a^	74.8 ± 6.9^a^	68.9 ± 7.4	17.01	<.001
Education years	3.9 ± 4.0^a^	5.5 ± 4.6	6.7 ± 5.2	3.91	.022
CDR	1.2 ± 0.8^a^^,^ b	0.6 ± 0.3^a^	0.4 ± 0.4	24.88^c^	<.001
K‐GDS	13.4 ± 7.1	14.0 ± 7.2	14.5 ± 7.0	0.23	.795
CERAD‐K cognitive tests					
Total scores	28.7 ± 12.6^a^^,^ b	42.5 ± 10.7^a^	64.7 ± 11.8	86.17	<.001
MMSE	13.7 ± 5.6^a^^,^ b	19.9 ± 5.0^a^	24.4 ± 4.2	45.55	
Constructional praxis	6.4 ± 2.5^a^	7.9 ± 2.6	9.1 ± 1.7	11.71	
Word list memory	6.2 ± 4.4^a^^,^ b	9.6 ± 3.6^a^	16.7 ± 4.3	63.07	
Word list recall	0.5 ± 1.0^a^^,^ b	1.7 ± 1.8^a^	5.7 ± 1.9	98.57^c^	
Word list recognition	3.1 ± 3.0^a^^,^ b	5.2 ± 2.9^a^	9.2 ± 1.0	106.65^c^	
Verbal fluency	6.7 ± 4.0^a^^,^ b	10.0 ± 3.3^a^	13.9 ± 3.8	36.28	
Boston naming test	5.6 ± 2.9^a^^,^ b	8.1 ± 2.9^a^	11.1 ± 2.5	34.46	

AD, Alzheimer's dementia; K‐GDS, Korean Geriatric Depression Scales; MCI, Mild cognitive impairment; HC, Healthy control; CDR, Clinical Dementia Rating; MMSE, Mini‐Mental State Examination.

As for the scores of CERAD‐K cognitive test and K‐GDS, complete data were available for 38 AD patients, 64 MCI patients, and 30 HCs, and one or more data were not available for the remaining subjects.

a Indicates significance compared to HC group (p < .05).

b Indicates significance compared to MCI group (p < .05).

c Welch's ANOVA with Games‐Howell post hoc test. Otherwise, ANOVA with Tukey's post hoc test.

Figure S1 shows the MSEP of the three groups’ PSLR models using CERAD‐K total scores as a dependent variable, respectively. According to these plots, the optimal numbers of LVs were 1, 1, and 4 for the AD, MCI, and HC groups, respectively. In the case of the PLSR models using scores of CERAD‐K 7 subdomains and K‐GDS as dependent variables, the optimal numbers of LVs were all 1 for all three groups. For the AD, MCI, and HC groups, the selected LV number(s) explained 59.7%, 43.2%, and 94.0% of the variance for the CERAD‐K total scores, respectively. For the other dependent variables and predictors, the relevant information is presented in Table S1. As a whole, the CERAD‐K total score was the highest explained dependent variable, and the MMSE score was the second‐highest explained variable, whereas the K‐GDS score was the least‐explained variable.

**Figure 1:**
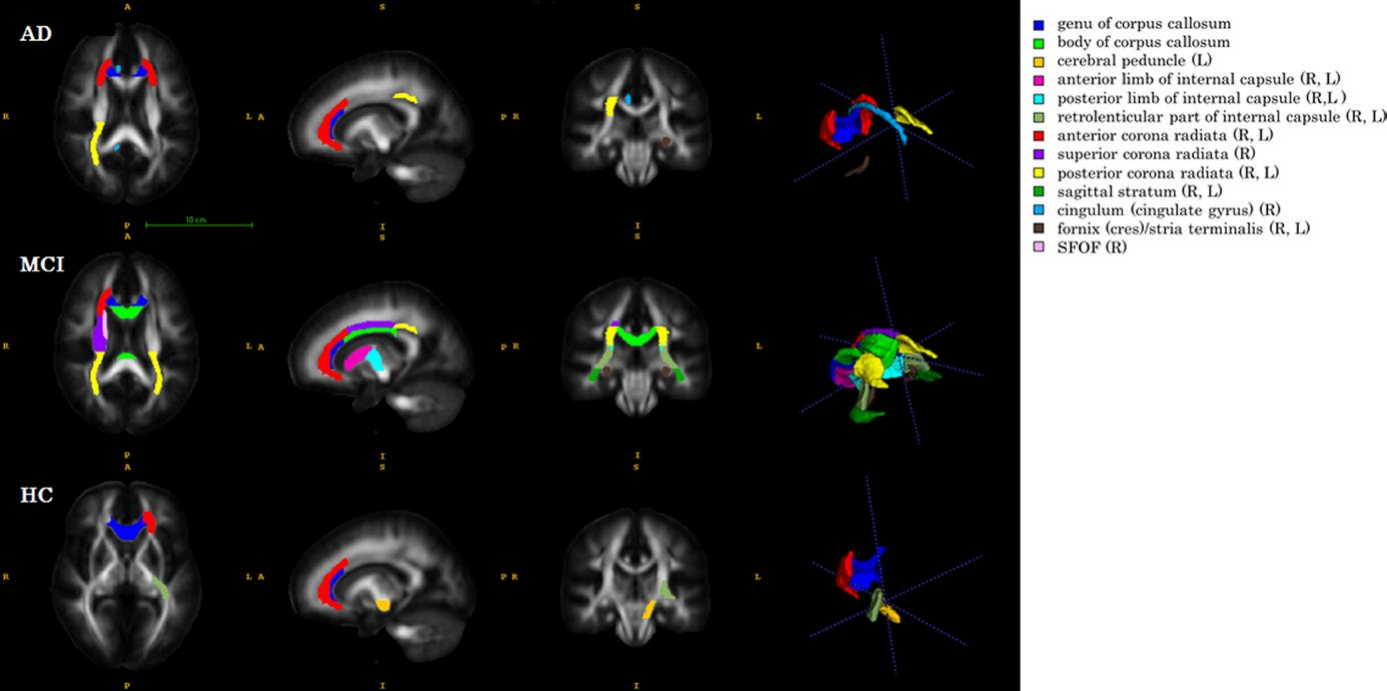
Significant fiber tracts obtained by thresholding p‐values <0.005 to the coefficients of three groups’ PLSR models using CERAD‐K total scores as dependent variables

Table 2 shows the most important hippocampal subfields and fiber tracts that are significantly (*p* < .005) correlated with the LV(s) with regard to the CERAD‐K total scores of each group. As shown in Table 2, the volumes of the hippocampal subfields, including CA1, CA2/3, CA4, molecular layer, subiculum, parasubiculum, hippocampal fissure, hippocampal tail, and GC‐ML‐DG, were significant predictors, although the global FA/MD values, (i.e., average of all regional FA/MD values) and several FA/MD values of regional fiber tracts, such as the anterior corona radiata, were also important predictors related to CERAD‐K total scores in the AD patients. In contrast with the AD patients, the FA/MD values were only significant contributors to the selected LV(s) in MCI patients and healthy controls. As shown in Figure 1, the important fiber tracts of the MCI patients were anterior/posterior limb and retrolenticular part of the internal capsule, genu of the corpus callosum, fornix (cres)/stria terminalis, sagittal stratum, posterior corona radiata, superior fronto‐occipital fasciculus (SFOF), etc., while important fiber tracts of healthy controls were cerebral peduncle, genu of corpus callosum, etc. Similar to the CERAD‐K total scores, the hippocampal subfield volumes were the main predictors of the scores for the CERAD‐K subdomains, including MMSE, Word list memory, Verbal fluency, etc. in AD patients (See Table S2), while the FA/MD values were main predictors for the scores of the CERAD‐K subdomains in MCI patients and healthy controls (See Tables S3 and S4). There was no significant predictor for the K‐GDS scores.

**Table 2:** Hippocampal subfields and fiber tracts that are significantly (p < .005) correlated with the latent variable(s) with regard to CERAD‐K total scores in three groups

Alzheimer's disease	reg.coef.	*t*‐value	*p*‐value	Mild cognitive impairment	reg.coef.	*t*‐value	*p*‐value
***Hippocampal subfields***				***FA***			
CA1 (L)	.0221	5.31	.0005	Anterior limb of internal capsule (L)	.0189	5.93	.0002
Hippocampal tail (L)	.0149	4.85	.0009	Genu of corpus callosum	.0176	4.86	.0009
Whole hippocampus (L)	.0228	4.66	.0012	SFOF (R)	.0203	4.82	.0009
CA1 (R)	.0216	4.57	.0013	Retrolenticular part of internal capsule (L)	.0136	4.42	.0017
Molecular layer (L)	.0233	4.41	.0017	Fornix (cres)/stria terminalis (R)	.0156	4.40	.0017
Whole hippocampus (R)	.0212	4.40	.0017	Anterior limb of internal capsule (R)	.0194	3.95	.0033
Subiculum (R)	.0222	4.35	.0019	***MD***			
Molecular layer (R)	.0219	4.30	.0020	Retrolenticular part of internal capsule (L)	−.0177	−6.62	.0001
GC‐ML‐DG (L)	.0217	4.08	.0027	Fornix (cres)/stria terminalis (L)	−.0251	−6.35	.0001
CA4 (L)	.0218	4.08	.0028	Sagittal stratum (L)	−.0175	−6.14	.0002
CA2/3 (L)	.0198	3.94	.0034	Posterior corona radiata (R)	−.0220	−5.84	.0002
Hippocampal.fissure (R)	.0207	3.86	.0038	Anterior limb of internal capsule (L)	−.0195	−5.47	.0004
Parasubiculum (L)	.0184	3.70	.0049	Posterior limb of internal capsule (R)	−.0149	−5.10	.0006
***FA***				Fornix (cres)/stria terminalis (R)	−.0228	−4.48	.0015
FA global	.0132	4.95	.0008	Anterior corona radiata (R)	−.0202	−4.35	.0018
Anterior corona radiata (L)	.0187	4.36	.0018	Genu of corpus callosum	−.0204	−4.35	.0018
Genu of corpus callosum	.0177	4.25	.0021	Superior corona radiata (R)	−.0172	−4.23	.0022
Posterior corona radiata (R)	.0134	3.85	.0039	Posterior limb of internal capsule (L)	−.0186	−4.20	.0023
***MD***				Retrolenticular part of internal capsule (R)	−.0160	−4.14	.0025
MD global	−.0191	−4.32	.0019	Sagittal stratum (R)	−.0242	−3.87	.0038
Fornix (cres)/stria terminalis (L)	−.0185	−4.23	.0022	Body of corpus callosum	−.0162	−3.86	.0038
Anterior corona radiata (R)	−.0192	−3.96	.0033	Posterior corona radiata (L)	−.0180	−3.86	.0039
Cingulum (cingulate gyrus) (R)	−.0199	−3.84	.0039	Anterior limb of internal capsule (R)	−.0181	−3.81	.0041
**Healthy controls**				***MD***			
***FA***				Genu of corpus callosum^1^	−.0178	−3.98	.0032
Cerebral peduncle (L)^2^	−.0730	−4.14	.0025	Anterior corona radiata (L)^1^	−.0146	−3.86	.0039
Cerebral_peduncle (L)^3^	−.0702	−3.91	.0036	Retrolenticular part of internal capsule (L)^1^	−.0203	−3.79	.0043

CA, Cornu Ammonis; FA, fractional anisotropy; MD, mean diffusivity; GC‐ML‐DG, granule cell layer‐molecular layer‐dentate gyrus; SFOF, superior fronto‐occipital fasciculus.

Superscripts in healthy controls indicate 1st to 3rd latent variables.

**Figure 2:**
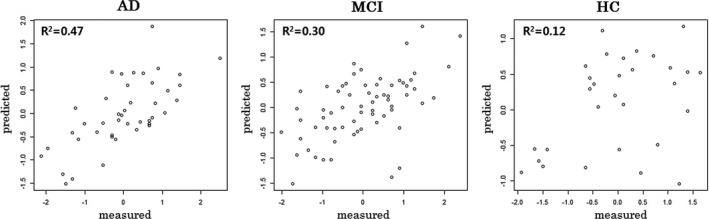
Predicted versus measured CERAD‐K total scores of three groups. Predicted values are obtained by 10‐fold cross‐validation in each PLSR model

As shown in Figure 2, the PLSR model of patients with AD or MCI significantly predicted the CERAD‐K total scores in 10‐fold cross‐validation (AD: *R* ^2^ = .47, coefficient = .50, *t*‐value = 6.08, *p* = 3.32E‐07; MCI: *R* ^2^ = .30, coefficient = .35, *t*‐value = 5.29, *p* = 1.60E‐06), while the PLSR model of the HCs did not predict the CERAD‐K total scores (*R* ^2^ = .12, coefficient = .22, *t*‐value = 1.98, *p* = .057). In addition to the CERAD‐K total scores, the scores of five or six CERAD‐K subdomains, such as MMSE, Word list memory, Verbal fluency etc., can also be predicted using the PLSR model of patients with AD or MCI, respectively (for more information, see Table S5).

Finally, a total of 300 iterations of the linear regression between the predicted and observed values from the PLSR models, using 2/3 for training and 1/3 as a test set, exhibited that only CERAD‐K total scores and Verbal fluency can be significantly predicted using the PLSR models in patients with AD (CERAD‐K total scores: *R* ^2^ = .51, coefficient = 1.09, *t*‐value = 3.74, *p* = .018; Verbal fluency: *R* ^2^ = .41, coefficient = 1.26, *t*‐value = 3.07, *p* = .046). The same iterative testing also revealed that the CERAD‐K total scores of the MCI patients can be predicted from the PLSR models of the MCI patients (*R* ^2^ = .38, coefficient = 1.02, *t*‐value = 3.58, *p* = .025), and that no dependent variables can be predicted from the PLSR models in the HCs (for more information, see Table S6).

## 4 DISCUSSION

In this study, we found that cognitive function of MCI and AD patients can be predicted using PLSR models in which the predictors are the hippocampal subfield volumes and DTI metrics (FA/MD). As for the MCI patients, DTI metrics were mostly significant predictors of cognitive function, whereas hippocampal subfield volumes were the main contributors to cognitive function in AD patients, although global FA/MD values were also significant predictors. The 10‐fold cross‐validation of the PLSR models of patients with AD or MCI showed that CERAD‐K total scores and scores of the several CERAD‐K subdomains can be significantly predicted, and further, stricter 300 iterative tests clearly proved that the global cognition measured by the CERAD‐K total scores can be reliably predicted using PLSR models with hippocampal volumes and DTI metrics in patients with AD or MCI.

As for the cognitive impairment of AD, Bozzali and colleagues (Bozzali et al., 2012) suggested that brain deafferentation through the cingulum is likely to play a remarkable role. In this hypothesis, neuronal loss of the medial temporal lobe at early stages of AD may lead to axonal loss, and then deafferentation of other brain regions, where these axons project, is considered to contribute to the cognitive disabilities in AD. On the other hand, the myelin model of the human brain proposed by Bartzokis (Bartzokis, 2011) considers AD as homeostatic responses to age‐related myelin breakdown, and a key protein of AD (i.e., Aβ and tau) is a byproduct of the myelin repair process rather than the primary cause of AD. In this model, the spread pattern of the AD lesion “recapitulates the myelination pattern in reverse” (Braak & Braak, 1996), that is, later‐myelinated neocortical projection fibers are affected first, while early‐myelinated large‐diameter from motor and sensory areas are affected least and last (Bartzokis, Lu, & Mintz, 2007) since the myelin sheaths are structurally more vulnerable when they are produced later during brain development (Amlien & Fjell, 2014).

Our findings imply that the myelin breakdown model is more plausible for pre‐ or early AD pathogenesis than the brain deafferentation model, since cognitive impairment of the MCI patients is predicted by the DTI metrics of cortico‐cortical association fibers like corpus callosum and corona radiata, while the brain deafferentation model is more readily explainable for cognitive impairment of late‐stage AD since AD patients’ cognitive disabilities are mainly influenced by hippocampal atrophy, MD of the cingulum, and so on. WM microscopic changes may occur before neuronal degradation and atrophy can be detectable on a macroscopic level (Muller et al., 2005). Accordingly, as other researchers have contended, DTI might be a more sensitive and quantifiable tool for early detection of AD than conventional MRI techniques (Neil, Miller, Mukherjee, & Huppi, 2002; Sun et al., 2005).

Our findings indicate that higher FA and lower MD values predicted higher cognitive functions in MCI or AD patients, and these are in agreement with previous studies reporting a significant association between decreased MMSE and reduced FA (Bai et al., 2009) or increased MD (Muller et al., 2007), either within or across groups. A recent meta‐analysis (Sexton et al., 2011) revealed that impaired global cognitive functions of AD patients were associated with an absolute effect size for FA in the parietal region while those of MCI patients were associated with an absolute effect size of FA in the frontotemporal and parietal regions and MD in the frontal region and corpus callosum. These findings suggest that in pre‐AD stages, diffuse WM alterations are more involved in cognitive functions than in AD stages, which is consistent with our results. It is plausible that myelin breakdown may result in a slow progression of the disruption in neural transmissions, which leads to the degradation of temporal synchrony in widely distributed neural networks. Such impaired synchronization of large‐scale neural networks may impair higher cognitive functions, including memory (Bartzokis, 2004).

In our study, the FA or MD value of the genu of corpus callosum was a significant predictor for global cognition in all three groups and for Word list memory in MCI patients, and the MD value of the body of corpus callosum was a significant predictor for global cognition in MCI patients. Atrophies and FA/MD changes in the anterior (genu) and the posterior (splenium) subregions of corpus callosum are widely accepted in AD (Sexton et al., 2011), and these alterations are suggested to be present in the early stages of AD (Di Paola, Spalletta, & Caltagirone, 2010). The genu receives axons directly from the prefrontal cortex and myelinates later than the splenium, which receives axons from temporo‐parietal regions that typically exhibit atrophy and hypometabolism in the AD (Ishii, 2014). Our findings that fiber tracts located in not the splenium but the genu predicted cognitive functions for MCI patients imply that the myelin breakdown model is more plausible than the Wallerian degeneration model in the early stage of AD pathogenesis.

Burgess and colleagues (Burgess, Maguire, & O'Keefe, 2002) mentioned that the right or left hippocampus has a different role according to the type of memory. For example, the right hippocampus is particularly involved in visuo‐spatial memory, with the left hippocampus more involved in episodic or autobiographical memory (Burgess et al., 2002). In our study, verbal memory and fluency are also largely predicted by the volume of the left hippocampal subfields, and this is compatible with a previous study reporting a significant association between the shrinkage of the left hippocampus and impaired verbal memory of AD (Laakso et al., 1995).

Meanwhile, a meta‐analysis of the relationship between memory performance and hippocampal volumes showed little evidence for the bigger‐is‐better hypothesis in older adults (Van Petten, 2004). In this analysis, studies showed extreme variability in the relationship between hippocampal size and episodic memory in older adults. However, in the case in which hippocampal atrophy becomes obvious with marked cognitive disabilities as AD progresses, the hippocampal volume itself seems to be an important biomarker to predict the degree of cognitive dysfunction, as our study implied. In our study, the CA1 volume was an important predictor of global cognition of AD patients. CA1 is known to be the first hippocampal area affected by neurofibrillary tangles, and it shows a maximal volume decrease (about 27%) in AD patients (La Joie et al., 2013; Schonheit, Zarski, & Ohm, 2004). Since CA1 and subiculum are the hippocampal subfields that show highly significant atrophy and neuronal loss in AD (West, Coleman, Flood, & Troncoso, 1994), follow‐up volumetry of these subfields will be useful to predict progression for MCI patients.

### 4.1 Limitations

There are several limitations of our study. First, a significant portion of the participants exhibited depressive symptoms since we did not exclude participants based on their K‐GDS scores. In one meta‐analysis of depression, patients exhibited about 8–10% reduced hippocampal volumes (Videbech & Ravnkilde, 2004). Thus, it is possible that the depressive symptoms of the subjects may affect the hippocampal volumes and general cognitive functions. However, depression is very common, and 30–40% of patients with AD or MCI have comorbid depression (Chi et al., 2015; Ismail et al., 2015). Therefore, our prediction model has a strength in that it can be generalized to a clinical situation. Second, predictor variables were not corrected by ICV or age, thus the whole brain volume as well as hippocampal atrophy and age‐related WM alterations may have influenced the evaluation of the importance of predictors. Third, only female subjects were included in our analysis, which limits the generalizability of our findings. Last but not least, a substantial portion of the patients with MCI will not progress to AD, so DTI metrics‐cognition associations in MCI patients should not be interpreted as equal to findings of prodromal AD, which warrants further longitudinal follow‐up research.

## 5 CONCLUSION

Our findings show different structural contributions to cognitive function between MCI and AD patients and imply that diffuse microstructural changes in WM may precede hippocampal atrophy during AD neurodegenerative processes. Our study also clearly showed that the cognitive function of MCI and AD patients can be predicted using hippocampal subfield volumetry and DTI metrics. In the future, a more objective evaluation will be possible on cognitive function and disease progression in patients with MCI or AD.

## CONFLICT OF INTEREST

None declared.
