# Supplementary figures and images for: Predicting neurocognitive function with hippocampal volumes and DTI metrics in patients with Alzheimer's dementia and mild cognitive impairment
Source: Brain Behav. 2017 Jul 30;7(9):e00766. doi: 10.1002/brb3.766 (PMC5607539; doi:10.1002/brb3.766)

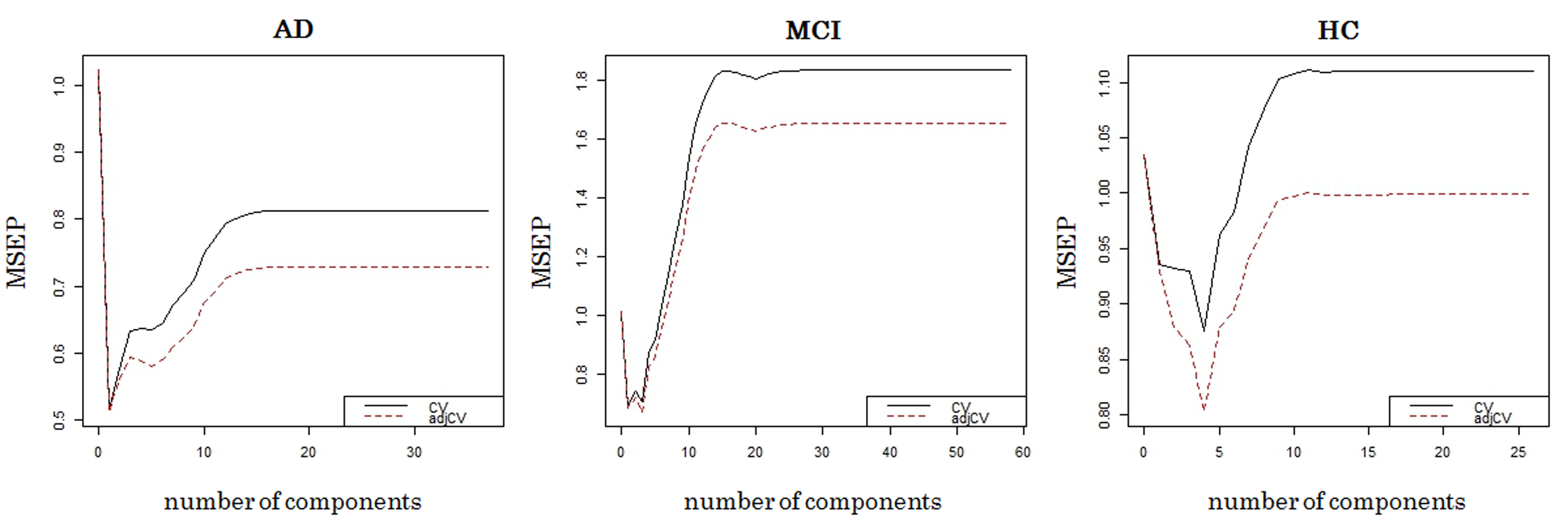

Supplement: Supplementary file 1 [file BRB3-7-e00766-s001.tif]
