# Supplementary material for: Predicting neurocognitive function with hippocampal volumes and DTI metrics in patients with Alzheimer's dementia and mild cognitive impairment
Source: Brain Behav. 2017 Jul 30;7(9):e00766. doi: 10.1002/brb3.766 (PMC5607539; doi:10.1002/brb3.766)

**Supplementary Figure 1:** MSEP plots for PLSR models using CERAD-K total scores as a Y-variable of three groups. CV (black-solid line) is the ordinary cross-validation estimate, and adjCV (red-dashed line) is a bias-corrected cross-validation estimate. According to these plots, the optimal numbers of latent variables are selected as 1, 1, and 4, for AD, MCI, HC groups, respectively.


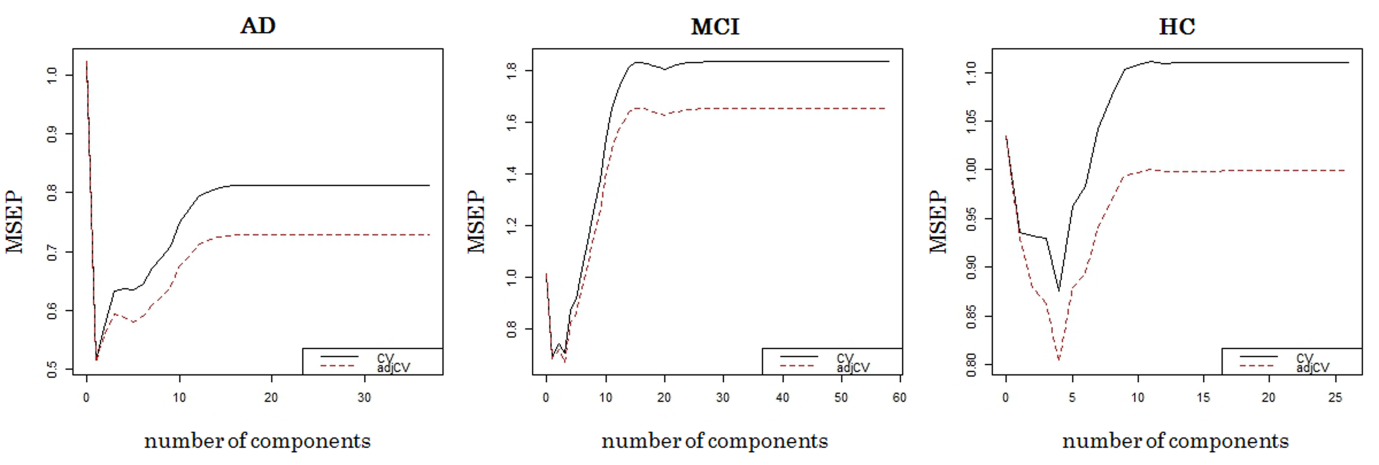

Supplement: Supplementary file 2 [file BRB3-7-e00766-s002.docx]
