# Supplementary material for: Predicting neurocognitive function with hippocampal volumes and DTI metrics in patients with Alzheimer's dementia and mild cognitive impairment
Source: Brain Behav. 2017 Jul 30;7(9):e00766. doi: 10.1002/brb3.766 (PMC5607539; doi:10.1002/brb3.766)
Supplement: Supplementary file 3 [file BRB3-7-e00766-s003.docx]

Supplementary Table 1: % variance explained by the selected number of latent variables.

|  | AD | MCI | HC |
| --- | --- | --- | --- |
| Independent variables | 22.1 | 22.3 | 27.0 |
| MMSE | 44.4 | 26.3 | 29.4 |
| Constructional praxis | 10.9 | 12.6 | 15.1 |
| Word list memory | 37.1 | 23.0 | 13.8 |
| Word list recall | 13.3 | 15.5 | 27.7 |
| Word list recognition | 22.7 | 19.0 | 12.3 |
| Verbal fluency | 43.5 | 18.9 | 15.7 |
| Boston naming test | 27.4 | 7.3 | 19.9 |
| K-GDS | 1.2 | 1.2 | 1.3 |

Supplementary Table 2: Hippocampal subfields and fiber tracts that are significantly (p<0.005) correlated with the latent variables with regard to the scores of 7 CERAD-K subdomains and K-GDS in patients with Alzheimer’s disease.

| **MMSE scores** | reg.coef. | t-value | | p-value | **Word list recognition** | reg.coef. | t-value | p-value |
| --- | --- | --- | --- | --- | --- | --- | --- | --- |
| ***Hippocampal subfields*** |  |  |  | | ***Hippocampal subfields*** |  |  |  |
| hippocampal tail (L)  CA2/3 (L)  GC-ML-DG (L)  CA4 (L)  whole hippocampus (L) | 0.0132  0.0180  0.0203  0.0204  0.0204 | 5.68  4.86  4.44  4.33  3.99 | | 0.0003  0.0009  0.0016  0.0019  0.0031 | hippocampal tail (L) | 0.0107 | 3.88 | 0.0037 |
|  |  |  |  |  | **Verbal fluency** |  |  |  |
|  |  |  |  |  | ***Hippocampal subfields*** |  |  |  |
|  |  |  |  |  | hippocampal tail (L)  CA2/3 (L)  CA4 (L)  GC-ML-DG (L)  whole hippocampus (L)  hippocampal fissure (L)  molecular layer (L) | 0.0144  0.0195  0.0222  0.0221  0.0221  0.0195  0.0228 | 8.96  4.67  4.22  4.20  4.11  3.88  3.77 | 8.88E-06 0.0012  0.0023  0.0023  0.0026  0.0038  0.0044 |
| ***FA*** |  |  | |  |  |  |  |  |
| posterior corona radiata (L) | 0.0084 | 3.86 | | 0.0038 |  |  |  |  |
| **Constructional praxis** |  |  | |  |  |  |  |  |
| none |  |  | |  |  |  |  |  |
| **Word list memory** |  |  | |  |  |  |  |  |
| ***Hippocampal subfields*** |  |  | |  | ***MD*** |  |  |  |
| hippocampal tail (L)  CA2/3 (L) | 0.0131  0.0178 | 4.71  3.86 | | 0.0011  0.0039 | retrolenticular part of internal capsule (R)  fornix (cres)/stria terminalis (L) | -0.0183  -0.0139 | -4.11  -3.92 | 0.0026  0.0035 |
| ***FA*** |  |  | |  | **Boston naming test** |  |  |  |
| posterior corona radiata (L) | 0.0083 | 4.10 | | 0.0027 | ***Hippocampal subfields*** |  |  |  |
| **Word list recall** |  |  | |  | hippocampal_tail (L) | 0.0117 | 3.69 | 0.0050 |
| none |  |  | |  | **K-GDS scores** |  |  |  |
|  |  |  | |  | none |  |  |  |

CA, Cornu Ammonis; GC-ML-DG, granule cell layer-molecular layer-dentate gyrus.

Supplementary Table 3: Hippocampal subfields and fiber tracts that are significantly (p<0.01) correlated with the latent variables with regard to the scores of 7 CERAD-K subdomains and K-GDS in patients with mild cognitive impairment.

| **MMSE scores** | reg. coef. | t-value | p-value | **Word list memory** | reg. coef. | t-value | p-value |
| --- | --- | --- | --- | --- | --- | --- | --- |
| ***Hippocampal subfields*** |  |  |  | ***FA*** |  |  |  |
| subiculum (R)  GC-ML-DG (R) | 0.0121  0.0137 | 4.07  3.71 | 0.0028  0.0049 | posterior corona radiata (R)  genu of corpus callosum  posterior corona radiata (L)  Anterior limb of internal capsule (L)  FA global  SFOF (R)  SFOF (L)  Tapetum (R) | 0.0123  0.0121  0.0082  0.0121  0.0105  0.0135  0.0109  0.0110 | 5.37  5.32  4.85  4.32  3.92  3.76  3.73  3.71 | 0.0005  0.0005  0.0009  0.0019  0.0035  0.0045  0.0047  0.0049 |
| ***FA*** |  |  |  |  |  |  |  |
| anterior limb of internal capsule (L)  fornix (cres)/stria terminalis (R)  SFOF (R)  SFOF (L) | 0.0130  0.0109  0.0145  0.0117 | 4.76  4.33  4.23  4.20 | 0.0010  0.0019  0.0022  0.0023 |  |  |  |  |
| ***MD*** |  |  |  |  |  |  |  |
| fornix (cres)/stria terminalis (L)  anterior limb of internal capsule (R)  sagittal stratum (R)  fornix (cres)/stria terminalis (R)  sagittal stratum (L)  anterior limb of internal capsule (L)  retrolenticular part of internal capsule (L)  SFOF (R)  Fornix column and body of fornix | -0.0184  -0.0114  -0.0167  -0.0155  -0.0142  -0.0132  -0.0143  -0.0106  -0.0133 | -6.68  -5.18  -4.80  -4.70  -4.67  -4.11  -3.99  -3.81  -3.73 | 0.0001  0.0006  0.0010  0.0011  0.0012  0.0026  0.0032  0.0041  0.0047 | ***MD*** |  |  |  |
|  |  |  |  | anterior limb of internal capsule (L)  fornix column and body of fornix  posterior corona radiata (R)  retrolenticular part of internal capsule (L)  sagittal stratum (R)  SFOF (R)  fornix (cres)/stria terminalis (L)  posterior thalamic radiation (R)  posterior limb of internal capsule (L)  superior corona radiata (L)  fornix (cres)/stria terminalis (R)  anterior limb of internal capsule (R)  posterior corona radiata (L) | -0.0123  -0.0124  -0.0167  -0.0133  -0.0155  -0.0098  -0.0171  -0.0110  -0.0114  -0.0114  -0.0144  -0.0106  -0.0126 | -5.68  -4.94  -4.48  -4.26  -4.17  -3.94  -3.87  -3.83  -3.81  -3.76  -3.75  -3.72  -3.69 | 0.0003  0.0008  0.0015  0.0021  0.0024  0.0034  0.0038  0.0040  0.0041  0.0045  0.0046  0.0048  0.0050 |
| **Constructional praxis** |  |  |  |  |  |  |  |
| ***MD*** |  |  |  |  |  |  |  |
| anterior limb of internal capsule (R)  fornix (cres)/stria terminalis (L) | -0.0080  -0.0128 | -3.87  -3.72 | 0.0038  0.0048 |  |  |  |  |
| **Word list recognition** |  |  |  |  |  |  |  |
| ***Hippocampal subfields*** |  |  |  | **Word list recall** |  |  |  |
| molecular layer (R)  presubiculum (R)  GC-ML-DG (R)  whole hippocampus (R)  subiculum (R)  CA4 (R) | 0.0108  0.0092  0.0120  0.0108  0.0106  0.0109 | 4.55  4.38  4.26  3.99  3.99  3.78 | 0.0014  0.0018  0.0021  0.0031  0.0032  0.0044 | none |  |  |  |
|  |  |  |  | **Verbal fluency** |  |  |  |
|  |  |  |  | ***FA*** |  |  |  |
|  |  |  |  | retrolenticular part of internal capsule (L) | 0.0100 | 4.05 | 0.0029 |
|  |  |  |  | ***MD*** |  |  |  |
|  |  |  |  | retrolenticular part of internal capsule (L) | -0.0125 | -3.94 | 0.0034 |
| ***FA*** |  |  |  | **Boston naming test** |  |  |  |
| fornix (cres)/stria terminalis (R)  retrolenticular part of internal capsule (L) | 0.0095  0.0100 | 4.34  3.80 | 0.0019  0.0042 | none |  |  |  |
|  |  |  |  | **K-GDS scores** |  |  |  |
| ***MD*** |  |  |  | none |  |  |  |
| retrolenticular part of internal capsule (L)  fornix (cres)/stria terminalis (R) | -0.0125  -0.0136 | -4.23  -3.75 | 0.0022  0.0046 |  |  |  |  |

CA, Cornu Ammonis; GC-ML-DG, granule cell layer-molecular layer-dentate gyrus; HATA, hippocampus-amygdala-transition-area; SFOF, superior fronto-occipital fasciculus.

Supplementary Table 4: Hippocampal subfields and fiber tracts that are significantly (p<0.005) correlated with the latent variables with regard to the scores of 7 CERAD-K subdomains and K-GDS in healthy controls.

| **MMSE scores** | | reg. coef. | t-value | p-value | **Word list memory** | reg. coef. | t-value | p-value |
| --- | --- | --- | --- | --- | --- | --- | --- | --- |
| none | |  |  |  | none |  |  |  |
| **Constructional praxis** | |  |  |  | **Word list recall** |  |  |  |
| none | |  |  |  | ***FA*** |  |  |  |
| **Word list recognition** | |  |  |  | posterior corona radiata (L) | 0.0220 | 3.85 | 0.0039 |
| none | |  |  |  | ***MD*** |  |  |  |
| **Verbal fluency** | |  |  |  | posterior corona radiata (L)  posterior thalamic radiation (L)  fornix column and body of fornix  anterior corona radiata (R)  sagittal stratum (L)  retrolenticular part of internal capsule (L) | -0.0149  -0.0138  -0.0144  -0.0167  -0.0140  -0.0172 | -6.73  -5.88  -5.32  -4.83  -4.39  -3.80 | 0.0001  0.0002  0.0005  0.0009  0.0017  0.0042 |
| ***MD*** | |  |  |  |  |  |  |  |
| retrolenticular part of internal capsule (L) | | -0.0129 | -3.95 | 0.0033 |  |  |  |  |
| **Boston naming test** | |  |  |  |  |  |  |  |
| none |  | |  |  |  |  |  |  |
| **K-GDS scores** | |  |  |  |  |  |  |  |
| none | |  |  |  |  |  |  |  |

Supplementary Table 5: Parametric values of linear regression models describing relationships between predicted and observed dependent variables in the 10-fold cross-validated partial least squares regression.

|  | R^2^ | Residual std. error | Coefficient | | t-value | F-statistic | p-value |
| --- | --- | --- | --- | --- | --- | --- | --- |
|  |  |  | estimate | std. error |  |  |  |
| Alzheimer’s disease |  |  |  |  |  |  |  |
| CERAD-K total scores | 0.47 | 0.53 | 0.50 | 0.08 | 6.08 | 36.98 | **3.32E-07** |
| MMSE | 0.26 | 0.48 | 0.31 | 0.09 | 3.63 | 13.16 | **<0.001** |
| Constructional praxis | 0.02 | 0.31 | 0.05 | 0.05 | 0.94 | 0.88 | 0.355 |
| Word list memory | 0.21 | 0.48 | 0.25 | 0.08 | 3.11 | 9.69 | **0.004** |
| Word list recall | 0.07 | 0.20 | 0.09 | 0.05 | 1.62 | 2.61 | 0.114 |
| Word list recognition | 0.12 | 0.41 | 0.15 | 0.06 | 2.27 | 5.14 | **0.029** |
| Verbal fluency | 0.33 | 0.48 | 0.33 | 0.08 | 4.24 | 17.98 | **<0.001** |
| Boston naming test | 0.11 | 0.46 | 0.16 | 0.07 | 2.16 | 4.68 | **0.037** |
| K-GDS | 0.23 | 0.13 | -0.07 | 0.02 | -3.29 | 10.84 | **0.002** |
| Mild cognitive impairment | | | | | | | |
| CERAD-K total scores | 0.30 | 0.53 | 0.35 | 0.07 | 5.29 | 27.96 | **1.60E-06** |
| MMSE | 0.14 | 0.45 | 0.18 | 0.06 | 3.15 | 9.93 | **0.003** |
| Constructional praxis | 0.06 | 0.33 | 0.09 | 0.04 | 2.06 | 4.24 | **0.044** |
| Word list memory | 0.15 | 0.41 | 0.17 | 0.05 | 3.25 | 10.56 | **0.002** |
| Word list recall | 0.06 | 0.37 | 0.09 | 0.05 | 2.02 | 4.09 | **0.048** |
| Word list recognition | 0.10 | 0.40 | 0.13 | 0.05 | 2.58 | 6.64 | **0.012** |
| Verbal fluency | 0.13 | 0.38 | 0.15 | 0.05 | 3.08 | 9.51 | **0.003** |
| Boston naming test | 0.01 | 0.27 | 0.03 | 0.03 | 0.82 | 0.67 | 0.416 |
| K-GDS | 0.01 | 0.12 | -0.01 | 0.01 | -0.80 | 0.64 | 0.426 |
| Healthy controls |  |  |  |  |  |  |  |
| CERAD-K total scores | 0.12 | 7.14 | 0.22 | 0.11 | 1.98 | 3.93 | 0.057 |
| MMSE | 0.07 | 2.29 | 0.14 | 0.10 | 1.42 | 2.02 | 0.166 |
| Constructional praxis | <0.01 | 0.76 | -0.01 | 0.08 | -0.09 | 0.01 | 0.928 |
| Word list memory | 0.02 | 2.19 | 0.06 | 0.09 | 0.67 | 0.46 | 0.505 |
| Word list recall | 0.07 | 1.03 | 0.15 | 0.10 | 1.43 | 2.04 | 0.164 |
| Word list recognition | 0.04 | 0.39 | 0.07 | 0.07 | 1.04 | 1.08 | 0.308 |
| Verbal fluency | 0.07 | 1.90 | 0.14 | 0.09 | 1.47 | 2.15 | 0.154 |
| Boston naming test | <0.01 | 1.32 | 0.01 | 0.10 | 0.11 | 0.01 | 0.916 |
| K-GDS | 0.01 | 3.75 | -0.04 | 0.10 | -0.46 | 0.21 | 0.652 |

P-values <0.05 are presented in bold.

Supplementary Table 6: Average parametric values of a total of 300 linear regression models. Predictive performance of PLSR models are obtained by averaging 300 iterations.

|  | R^2^ | | Coefficient estimate | | t-value | | p-value | |
| --- | --- | --- | --- | --- | --- | --- | --- | --- |
|  | mean | SD | mean | SD | mean | SD | mean | SD |
| Alzheimer’s disease |  |  |  |  |  |  |  |  |
| CERAD-K total scores | 0.51 | 0.15 | 1.09 | 0.37 | 3.74 | 1.24 | **0.018** | 0.046 |
| MMSE | 0.30 | 0.14 | 1.06 | 0.44 | 2.46 | 0.92 | 0.076 | 0.118 |
| Constructional praxis | 0.14 | 0.12 | 1.48 | 1.93 | 1.26 | 0.95 | 0.323 | 0.278 |
| Word list memory | 0.30 | 0.14 | 1.11 | 0.45 | 2.40 | 0.91 | 0.082 | 0.124 |
| Word list recall | 0.10 | 0.08 | 1.60 | 1.25 | 1.11 | 0.58 | 0.346 | 0.221 |
| Word list recognition | 0.23 | 0.15 | 1.25 | 0.79 | 1.92 | 0.97 | 0.166 | 0.202 |
| Verbal fluency | 0.41 | 0.16 | 1.26 | 0.64 | 3.07 | 1.12 | **0.046** | 0.109 |
| Boston naming test | 0.27 | 0.15 | 1.14 | 0.65 | 2.18 | 0.98 | 0.125 | 0.178 |
| K-GDS | 0.06 | 0.09 | 0.19 | 23.40 | -0.37 | 0.90 | 0.559 | 0.282 |
| Mild cognitive impairment | | | |  |  |  |  |  |
| CERAD-K total scores | 0.38 | 0.16 | 1.02 | 0.37 | 3.58 | 1.30 | **0.025** | 0.065 |
| MMSE | 0.25 | 0.15 | 1.08 | 0.58 | 2.53 | 1.18 | 0.098 | 0.173 |
| Constructional praxis | 0.14 | 0.11 | 1.26 | 0.90 | 1.75 | 0.87 | 0.185 | 0.206 |
| Word list memory | 0.22 | 0.13 | 1.08 | 0.52 | 2.33 | 0.97 | 0.093 | 0.147 |
| Word list recall | 0.16 | 0.15 | 1.15 | 2.71 | 1.67 | 1.41 | 0.269 | 0.300 |
| Word list recognition | 0.15 | 0.12 | 0.91 | 0.61 | 1.75 | 1.03 | 0.200 | 0.242 |
| Verbal fluency | 0.21 | 0.13 | 1.29 | 0.78 | 2.23 | 0.98 | 0.113 | 0.163 |
| Boston naming test | 0.09 | 0.08 | 1.46 | 1.23 | 1.27 | 0.78 | 0.299 | 0.265 |
| K-GDS | 0.06 | 0.08 | -3.90 | 80.71 | -0.17 | 1.13 | 0.482 | 0.291 |
| Healthy controls |  |  |  |  |  |  |  |  |
| CERAD-K total scores | 0.23 | 0.19 | 0.71 | 0.52 | 1.47 | 1.07 | 0.292 | 0.259 |
| MMSE | 0.19 | 0.15 | 1.04 | 1.29 | 1.39 | 0.84 | 0.296 | 0.247 |
| Constructional praxis | 0.16 | 0.18 | 1.14 | 1.62 | 0.86 | 1.27 | 0.411 | 0.288 |
| Word list memory | 0.12 | 0.13 | 0.93 | 2.00 | 0.61 | 1.01 | 0.466 | 0.277 |
| Word list recall | 0.15 | 0.15 | 0.63 | 0.89 | 0.91 | 1.03 | 0.413 | 0.295 |
| Word list recognition | 0.16 | 0.16 | 3.32 | 36.98 | 1.03 | 0.96 | 0.404 | 0.296 |
| Verbal fluency | 0.16 | 0.16 | 1.09 | 1.09 | 1.09 | 0.97 | 0.396 | 0.289 |
| Boston naming test | 0.15 | 0.15 | 1.03 | 1.38 | 1.06 | 0.92 | 0.406 | 0.292 |
| K-GDS | 0.05 | 0.07 | 0.14 | 21.87 | -0.13 | 0.72 | 0.632 | 0.250 |

After data were randomly split into 2/3 and 1/3 for training and test set, CERAD-K and K-GDS scores of test set were predicted with a PLSR model using training set, and a linear regression was attempted to model the relationship between observed and predicted scores (a total of 300 iterative testing).
